# Pharmaceutical enterprises drug quality strategy *Moran* analysis considering government supervision and new media participation

**DOI:** 10.3389/fpubh.2022.1079232

**Published:** 2023-01-17

**Authors:** Yanping Xu, Lilong Zhu

**Affiliations:** ^1^School of Business, Shandong Normal University, Ji'nan, China; ^2^Quality Research Center, Shandong Normal University, Ji'nan, China

**Keywords:** drug quality improvement strategy, drug cost reduction strategy, government supervision, new media participation, *Moran process*

## Abstract

The improvement of drug quality requires not only the supervision of government, but also the participation of new media. Therefore, this paper considers the impact of government regulation and new media reports on pharmaceutical enterprises, constructs a *Moran Process* evolutionary game model, and analyzes the evolution trajectory of pharmaceutical enterprises' choice of drug quality improvement strategy and drug cost reduction strategy. We obtain the conditions for the two strategies to achieve evolutionary stability under the dominance of external factors and the dominance of expected returns. To verify the theoretical results, we conduct a numerical simulation by the software *MATLAB 2021b*. The results show that, first of all, when the government penalty is high, the drug quality improvement strategy tends to become an evolutionary stable solution, increasing the penalty amount will help promote the improvement of drug quality. What's more, when the government penalty is low and the new media influence is low, the drug cost reduction strategy is easier to dominate. The higher the new media influence, the higher the probability that pharmaceutical enterprises choose the drug quality improvement strategy. Thirdly, when the number of pharmaceutical enterprises is lower than a threshold, the drug quality improvement strategy is easier to dominate. Finally, the drug quality improvement strategy is dominant when the quality cost factor is low and the government penalty is high, the drug cost reduction strategy is dominant when the quality cost factor is high and the government penalty is low. Above all, this paper provides countermeasures and suggestions for the drug quality improvement of pharmaceutical enterprises in practice.

## 1. Introduction

In recent years, drug quality is related to people's life and health, economic development, and social stability, and it is the focus of public opinion. Many drug safety incidents occur frequently, such as, the incident of “vitamin C Yinqiao tablet containing poison” in 2013 (*www.ema.europa.eu*, 2013), the asbestos carcinogen incident of “Johnson & Johnson” in 2019 (*www.FDA.gov*, 2019), the fatal incident of “heparin sodium” in 2020 (*www.FDA.gov*, 2020).

These incidents have attracted the attention of government departments. In 2021, the World Health Organization (WHO) issued guidance on good supervisory practices and good trust practices to improve drug supervision efficiency. The guidance aims to effectively supervise drug through good supervisory practice and to promote greater collaboration between regional and national supervisory agencies (*www.WHO.int*, 2021). The US Food and Drug Administration (FDA) supports the media and other social forces to participate in drug supervision. Through the establishment of a drug supervision model in which the government, the market, and society cooperate, the FDA can effectively promote scientific supervision and protect public health (*www.FDA.gov*, 2017). The European Drug Agency (EMA) puts patients and medical institutions at the center of the supervisory system to ensure timely access to high-quality drug for patients. By collaborating with academic research institutes, the EMA establishes new supervisory approaches and innovation platforms to create a more adaptive supervisory system (*http://www.ema.europa.eu/*, 2019). To improve drug supervision efficiency, the Chinese government has issued a series of policies to urge pharmaceutical enterprises to implement their main responsibilities. These policies are conducive to strengthening the daily supervision of drug supervisory authorities and improving drug quality (*www.nmpa.gov.cn*, 2016).

With the development of the Internet, there are more and more channels for the public to participate in drug supervision through new media. New media has become an important participator in drug quality supervision. The supervision of new media not only promotes the resolution of drug safety incidents, but also has a great impact on the choice of pharmaceutical enterprises' production strategy.

Therefore, based on the government supervision, this paper considers the drug quality strategic choice for pharmaceutical enterprises with new media participation. By constructing the *Moran process* stochastic evolutionary game model, the following three problems are solved. Firstly, how do the number of pharmaceutical enterprises in the market, the intensity of government penalties, and the participation of new media affect the drug quality strategic choice? Secondly, what is the difference between the drug quality strategic choice under the dominance of external factors and the dominance of expected returns? Thirdly, how do pharmaceutical enterprises that pursue different market shares choose in different condition?

The rest of this paper is arranged as follows, part 2 sorts out and reviews the relevant literature; part 3 puts forward hypotheses and builds the *Moran process* model for the drug quality strategic choice of pharmaceutical enterprises; part 4 is the stochastic evolution dynamics process of pharmaceutical enterprises *Moran* strategy; part 5 analyzes how pharmaceutical enterprises choose drug quality improvement strategy and drug cost reduction strategy under the dominance of external factors and the dominance of expected returns; part 6 uses *MATLAB 2021b* to simulate the strategic choice process of pharmaceutical enterprises; part 7 is the discussions and part 8 is the conclusions.

## 2. Literature review

### 2.1. Drug quality

Drug quality is one of the key issues of concern around the world, and it is mainly affected by information asymmetry between manufacturers and consumers (1), low levels of enterprises' social responsibility, and insufficient government supervision. Drug quality levels should be matched to the most pressing needs of the public (2) and drug quality requires specific technical improvements (3). Improving the transparency and accountability of pharmaceutical enterprises (4), introducing new drug production models (5), and implementing digital transformation strategies (6) can effectively improve drug quality. Drug quality supervision by government departments and third-party testing agencies can minimize the probability of illegal production by pharmaceutical enterprises and reduce the circulation of the inferior drug (7).

### 2.2. Government supervision

Government departments have the responsibility to supervise the pharmaceutical enterprises' production activities and drug quality to protect public health (8). Drug production is a long and complex process, and drug supervision is extremely necessary and useful (9). The lack of effective supervision leads to an unsafe drug supply (10). The punishment of government departments has an impact on the economic behavior of pharmaceutical enterprises (11). By increasing strict supervision (12), setting up a reasonable reward and punishment mechanism (13), and encouraging consumers to participate in quality supervision work (14), government departments can effectively promote the high-quality development of the pharmaceutical industry. At present, few countries have a complete supervision system, which leads to repeated incidents of the substandard drug (15).

### 2.3. New media participation

With the development of the Internet, the participation of new media in drug quality supervision as a third-party force has received extensive attention. The emergence of new media enables the public to take an open position on specific issues (16), making the delivery of drug information more rapid, extensive and transparent. By exposing product quality problems, the new media arouses social attention and urges local governments to strictly supervise (17). However, it can influence social perceptions according to subjective wishes when new media provides real-time information (18). The new media conspires with illegal pharmaceutical enterprises to obtain benefits, which leads to the public obtaining false information. False news spreads faster and farther on social media sites (19). New media platforms must do more to police their networks and reduce disinformation (20).

### 2.4. Moran process

The *Moran process* analysis the evolutionary dynamics of individual selection strategies (21). In the frequency-dependent Moran process, the strategic choice is judged by comparing the individual's returns with the returns of other individuals (22). When individuals using the new strategy start, the group supports invasion and replacement through a reciprocal strategy (23). The evolutionary game of finite groups is widely used in sociology, economics, management, and other fields. For example, it is used to predict how strategies in the player group evolve (24), and how to promote polluting enterprises to deal with emissions (25). The *Moran process* can promote cooperation between enterprises (26), and when the strength of selection among groups is strong enough, cooperation can be sustained (27).

To sum up, the existing literature still lacks a comprehensive consideration of government supervision and new media participation on the pharmaceutical enterprises' drug quality strategic choice. Therefore, compared with previous studies, this paper is mainly different in the following three aspects, firstly, based on the government supervision, we consider the choice of drug quality improvement strategy and drug cost reduction strategy of new media participation, and construct the stochastic evolutionary game model of *Moran process* of the pharmaceutical enterprises' drug quality strategy; secondly, we compare and analyze the difference between the pharmaceutical enterprises' strategic choice under the dominance of external factors and the dominance of expected returns, and calculate the conditions for the two strategies to take root; thirdly, we use *MATLAB 2021b* to simulate the pharmaceutical enterprises' strategic choice under different conditions, and puts forward countermeasures and suggestions for improving drug quality based on the analysis results. Through theoretical analysis and case analysis, we draw important conclusions such as the dominant conditions for pharmaceutical enterprises to adopt the “drug quality improvement strategy” and the critical scale of pharmaceutical enterprises for strategy transformation.

## 3. Model hypotheses

To analyze the drug quality strategic choice of pharmaceutical enterprises with government supervision and new media participation, we make the following hypotheses.

*H1* The feasible strategic choice of pharmaceutical enterprises is {drug quality improvement strategy, drug cost reduction strategy}, denoted as {*Q, L*}, drug cost reduction strategy may lead to a decrease in drug quality; the feasible strategic choice of new media is {true report, false report}, denoted as {*R, NR*}. *N* pharmaceutical enterprises take part in the *Moran process* (*N*≥2).

*H2* When pharmaceutical enterprises choose the drug cost reduction strategy, the drug production cost is *C*. When pharmaceutical enterprises choose the drug quality improvement strategy, the drug quality improvement level is *q*, so the drug quality improvement cost is (1+*q*) *C*. The pharmaceutical enterprises' drug selling income is *R*.

*H3* New media reports need to pay a cost *C*_*N*_. The true report brings value *V*_1_ to the new media, and the false report brings value *V*_2_. The true report brings positive effects to new media, so *V*_1_>*V*_2_.

*H4* The impact of the new media reports is η, η∈[0, 1]. When the real reports of the new media, the pharmaceutical enterprises that choose the drug quality improvement strategy get a positive impact *D*_*e*_, and the pharmaceutical enterprises that choose the drug cost reduction strategy get a negative impact *D*_*f*_. When two pharmaceutical companies choose the same strategy, the new media reports have no any impact.

*H5* Government departments supervise the drug quality. Pharmaceutical enterprises that choose the drug cost reduction strategy are punished by government departments, and the penalty is *F*.

The related parameters and descriptions are shown in Table 1.

**Table 1:** Related parameters and descriptions.

**Parameters**	**Descriptions**
*q*	Drug quality improvement level
*C*	Drug production costs when a drug cost reduction strategy is selected
*R*	Pharmaceutical enterprises' drug selling income
*N*	The number of pharmaceutical enterprises in the group
*C*_*N*_	The cost of new media report
*V*_1_	The value of true report in new media
*V*_2_	The value of false report in new media
η	The impact level of the new media reports
*D*_*e*_	The positive impact of choosing a drug quality improvement strategy
*D*_*f*_	The negative impact of choosing a drug cost reduction strategy
*F*	Penalties for enterprises that choose a drug cost reduction strategy

Based on the above hypotheses, this paper constructs the drug quality strategy game matrix jointly participated by pharmaceutical enterprises and new media, as shown in Table 2.

**Table 2:** Drug quality strategy game matrix.

**Pharmaceutical enterprises**	**New media**	
	**True report**	**False report**
Drug quality improvement strategy	*R*−(1 + *q*)*C* + η*D*_*e*_, *V*_1_−*C*_*N*_	*R*−(1 + *q*)*C*, *V*_2_−*C*_*N*_
Drug cost reduction strategy	*R*−*C*−*F*−η*D*_*f*_, *V*_1_−*C*_*N*_	*R*−*C*−*F*, *V*_2_−*C*_*N*_

According to the game matrix, when (*qC*−η*D*_*e*_) ≤ (*F* + η*D*_*f*_), the cost of drug quality improvement is less than the sum of government penalties and the negative effects of choosing drug cost reduction strategy. The pure strategy Nash equilibrium is {drug quality improvement strategy, true report}. When (*qC*−η*D*_*e*_)>(*F* + η*D*_*f*_), the pure strategy Nash equilibrium is {drug cost reduction strategy, true report}.

According to the homogeneity assumption of *N* pharmaceutical enterprises, the symmetrical returns matrix is obtained, as shown in Table 3.

**Table 3:** Symmetrical returns matrix of pharmaceutical enterprises.

**Pharmaceutical enterprises (G_1_)**	**Pharmaceutical enterprises (G**_**2**_ **)**	
	**Drug quality improvement strategy**	**Drug cost reduction strategy**
Drug quality improvement strategy	*R*−(1 + *q*)*C*, *R*−(1 + *q*)*C*	*R*−(1 + *q*)*C* + η*D*_*e*_, *R*−*C*−*F*−η*D*_*f*_
Drug cost reduction strategy	*R*−*C*−*F*−η*D*_*f*_, *R*−(1 + *q*)*C* + η*D*_*e*_	*R*−*C*−*F*, *R*−*C*−*F*

## 4. The *Moran* process analysis of pharmaceutical enterprises' drug quality strategy

The overall number of pharmaceutical enterprises in the market is *N*. The number of pharmaceutical enterprises that choose the drug quality improvement strategy is *i*, and the number of pharmaceutical enterprises that choose the drug cost reduction strategy is *N*−*i*. Expected benefits are the benefits generated by different strategic choice for pharmaceutical enterprises. According to the strategy returns matrix, the expected returns of pharmaceutical enterprises that choose the drug quality improvement strategy and the drug cost reduction strategy are,


(1) $$\begin{array}{c} \;\pi_{Q}^{i}=\pi_{QQ}+\pi_{QL}\\ =\frac{i-1}{N-1}\left[R-\left(1+q\right)C\right]+\frac{N-i}{N-1}\left[R-\left(1+q\right)C+\eta D_{e}\right]\\ \;i=1,2,\cdots \;,N-1 \end{array}$$

(2) $$\begin{array}{c} \;\pi_{L}^{i}=\pi_{LQ}+\pi_{LL}\\ =\frac{i}{N-1}\left[R-C-F-\eta D_{f}\right]+\frac{N-i-1}{N-1}\left[R-C-F\right]\\ \;i=1,2,\cdots \;,N-1 \end{array}$$

Among them, $\pi_{Q}^{i}$ is the expected returns of the pharmaceutical enterprise choosing the drug quality improvement strategy, and $\pi_{L}^{i}$ is the expected returns of the pharmaceutical enterprise choosing the drug cost reduction strategy.

In the market environment, external factors have an impact on the fitness function. For example, enterprises social responsibility and government policy. By introducing the selection strength ξ, ξ ∈[0, 1], the fitness linear function of the two strategies are obtained,


(3) $$\begin{array}{c} f_{i}=1-\xi +\xi \pi_{Q}^{i},g_{i}=1-\xi +\xi \pi_{L}^{i},\xi \in \left[0,1\right] \end{array}$$

In the *Moran process*, the probability of increasing a pharmaceutical enterprise that chooses the drug quality improvement strategy is $\frac{if_{i}}{if_{i}+\left(N-i\right)g_{i}}$. At each time, pharmaceutical enterprises make strategic adjustments. The transition probability matrix of the *Moran process* is tridiagonal, and the diagonal elements are,


(4) $$\begin{array}{c} Z_{i,i+1}=\frac{if_{i}}{if_{i}+\left(N-i\right)g_{i}}\times \frac{N-i}{N} \end{array}$$

(5) $$\begin{array}{c} Z_{i,i-1}=\frac{\left(N-i\right)f_{i}}{if_{i}+\left(N-i\right)g_{i}}\times \frac{i}{N} \end{array}$$

(6) $$\begin{array}{c} Z_{i,i}=1-Z_{i,i+1}-Z_{i,i-1} \end{array}$$

*Moran process* has two stable states, *i* = *N*, all pharmaceutical enterprises choose drug quality improvement strategy, *i* = 0, and all pharmaceutical enterprises choose drug cost reduction strategy. Next, the probability of rooting to the two states is calculated separately.

Let φ_0_ denote the probability that the number of pharmaceutical enterprises that choose the drug quality improvement strategy changes from *i* to *N*. It can be obtained from the total probability formula,


(7) $$\{\begin{array}{l} \varphi_{0}=0\\ \varphi_{i}=Z_{i,i-1}\varphi_{i-1}+Z_{i,i}\varphi_{i}+Z_{i,i+1}\varphi_{i+1},i=1,2,\cdots ,N-1\\ \varphi_{N}=1 \end{array}$$

Equation (4), (5), (6) are substituted into Equation (7), we can obtain,


(8) $$\begin{array}{c} \varphi_{i}=\varphi_{1}\left(1+\overset{i-1}{\underset{k=1}{\sum }}\overset{k}{\underset{n=1}{\prod }}\chi_{n}\right)=\frac{1+\overset{i-1}{\underset{k=1}{\sum }}\overset{k}{\underset{n=1}{\prod }}\frac{g_{i}}{f_{i}}}{1+\overset{N-1}{\underset{k=1}{\sum }}\overset{k}{\underset{n=1}{\prod }}\frac{g_{i}}{f_{i}}} \end{array}$$

If only one pharmaceutical enterprise chooses the drug quality improvement strategy in the initial state, the stable equilibrium probability that the final drug quality improvement strategy successfully occupies the entire market is,


(9) $$\begin{array}{c} \rho_{Q}=\varphi_{1}=\frac{1}{1+\overset{N-1}{\underset{k=1}{\sum }}\overset{k}{\underset{n=1}{\prod }}\frac{g_{i}}{f_{i}}} \end{array}$$

Conversely, if only one pharmaceutical enterprise chooses the drug cost reduction strategy in the initial state, the stable equilibrium probability that the final drug cost reduction strategy successfully occupies the entire market is,


(10) $$\begin{array}{c} \rho_{L}=1-\varphi_{N-1}=\frac{1}{1+\overset{N-1}{\underset{k=1}{\sum }}\overset{N-1}{\underset{i=k}{\prod }}\frac{f_{i}}{g_{i}}} \end{array}$$

When the mutation speed is relatively small, the strategy with a larger stable equilibrium probability maintains a high probability for a long period, and this strategy is more likely to become an evolutionary stable strategy.

## 5. Results analysis

### 5.1. Drug quality strategic choice under the dominance of external factors

In the decision-making process of pharmaceutical enterprises' drug quality strategy, the fitness function is not only affected by expected returns, but also affected by various external factors such as competitors' behavior and response, medical reform policies, laws and regulations, etc., which is the weak selection process.

In this situation, the selection strength is ξ → 0. We can obtain the Taylor formula expansion of Equation (9) and Equation (10) in ξ → 0.


(11) $$\begin{array}{c} \rho_{Q}=\frac{1}{1+\overset{N-1}{\underset{k=1}{\sum }}\overset{k}{\underset{n=1}{\prod }}\frac{g_{i}}{f_{i}}}\approx \frac{1}{N}\text{+}\frac{\xi }{\text{6N}}\left(\alpha \text{+}N\beta \right) \end{array}$$

(12) $$\begin{array}{c} \rho_{L}=\frac{1}{1+\overset{N-1}{\underset{k=1}{\sum }}\overset{N-1}{\underset{i=k}{\prod }}\frac{f_{i}}{g_{i}}}\approx \frac{1}{N}\text{+}\frac{\xi }{\text{6N}}\left(\gamma +N\delta \right) \end{array}$$

By calculating, we can get α = 3*qC* − *ηD*_*e*_ − 3*F* + *ηD*_*f*_, β = − 3*qC* + 2*ηD*_*e*_ + 3*F* + *ηD*_*f*_, γ = − 3*qC* − *ηD*_*e*_ + *ηD*_*f*_ + 3*F*, δ = 3*qC* − *ηD*_*e*_ − 2*ηD*_*f*_−3*F*.

Based on the research of Taylor et al. (28) and the stable equilibrium probability 1/*N*of pharmaceutical enterprises, the strategic choice of pharmaceutical enterprises is studied. When ρ_*Q*_ > 1/*N* and ρ_*L*_ < 1/*N*, the group supports the drug quality improvement strategy to replace the drug cost reduction strategy; when ρ_*Q*_ < 1/*N* and ρ_*L*_ > 1/*N*, the group supports the drug cost reduction strategy to replace the drug quality improvement strategy.

**Proposition 1** Under the condition of weak selection, when *qC* < (*F* + *ηD*_*f*_), if *D*_*f*_<*D*_*e*_, ρ_*Q*_ > 1/*N* is always established, and the group supports the drug quality improvement strategy to replace the drug cost reduction strategy.

***Proof*** ρ_*Q*_ > 1/*N* is equivalent to *y* = α + β*N* > 0. When *D*_*f*_<*D*_*e*_ and *qC* < (*F* + *ηD*_*f*_), $\frac{\partial y}{\partial N}\text{=}-3qC+2\eta D_{e}+3F+\eta D_{f}>0$, *y*(2) = −3*qC* + 3*F* + 3*ηD*_*e*_ + 3*ηD*_*f*_ > 0, when *N*≥2, *y* > 0 is always established.

Proposition 1 shows that when the cost of improving drug quality is lower than the sum of government penalties and negative effects for choosing drug cost reduction strategy, the pharmaceutical enterprises' strategic choice depends on the influence of new media reports. When the positive effect is high, pharmaceutical enterprises tend to choose drug quality improvement strategy.

**Proposition 2** When −3*qC* + 2*ηD*_*e*_ + 3*F* + *ηD*_*f*_ < 0, there is a threshold $N_{a}=\left[1+\frac{\eta D_{e}\text{+}2\eta D_{f}}{3qC-2\eta D_{e}-3F-\eta D_{f}}\right]$. If *N*∈[2, *N*_*a*_], we can get ρ_*Q*_ > 1/*N*, the drug quality improvement strategy is more likely to take root in the group. If *N*≥*N*_*a*_, we can get ρ_*Q*_ < 1/*N*, then the drug cost reduction strategy is more likely to take root in the group.

***Proof*** ρ_*Q*_ > 1/*N* is equivalent to *y* = α + β*N* > 0. When −3*qC* + 2*ηD*_*e*_ + 3*F* + *ηD*_*f*_ < 0, we get $\frac{\partial y}{\partial N}\text{=}-3qC+2\eta D_{e}+3F+\eta D_{f}<0$, the function is monotonically decreasing. Set *y* = α + β*N*_*a*_ = 0, we can get $N_{a}=1+\frac{\eta D_{e}+2\eta D_{f}}{3qC-\eta D_{e}-3F-\eta D_{f}}$. When *N*<*N*_*a*_, there is $\rho_{Q}>\frac{1}{N}$, and when*N*>*N*_*a*_, there is $\rho_{Q}<\frac{1}{N}$.

Proposition 2 shows that when the loss caused by the drug cost reduction strategy is low, there is a threshold *N*_*a*_ for the number of pharmaceutical enterprises. When the number of pharmaceutical enterprises is less than the threshold, pharmaceutical enterprises are willing to pay high quality improvement cost and tend to choose the drug quality improvement strategy.

### 5.2. Drug quality strategic choice under the dominance of expected returns

When pharmaceutical enterprises make strategic choice based on expected returns, it is a strong selection process, ξ = 1. In this situation, pharmaceutical enterprises are completely rational. Given the number of pharmaceutical enterprises that choose the drug quality improvement strategy at a certain moment, the choice preference of pharmaceutical enterprises can be judged by comparing the fitness function values of the two strategies at that moment. Suppose that,


(13) $$\begin{array}{c} h_{i}=f_{i}-g_{i},i=1,2,\cdots \;,N-1 \end{array}$$

Equations (1), (2), (3) are substituted into Equation (13), we can obtain,


(14) $$\begin{array}{c} h_{1}=f_{1}-g_{1}=-qC+\frac{\eta D_{f}}{N-1}+F+\eta D_{e} \end{array}$$

(15) $$\begin{array}{c} h_{N-1}=f_{N-1}-g_{N-1}=-qC+\frac{\eta D_{e}}{N-1}+F+\eta D_{f} \end{array}$$

If both *h*_1_ > 0 and *h*_*N* − 1_ > 0 are satisfied, the drug quality improvement strategy replaces the drug cost reduction strategy and evolves into a stable strategy. If both *h*_1_ < 0 and *h*_*N* − 1_ < 0 are satisfied, the drug cost reduction strategy replaces the drug quality improvement strategy and evolves into a stable strategy. If *h*_1_ > 0 and *h*_*N* − 1_ < 0, strategies cannot invade each other, two strategies exist in the group at the same time.

**Proposition 3** When *F* > *qC*, for all *N*≥2, there are *h*_1_ > 0 and *h*_*N* − 1_ > 0, the drug quality improvement strategy is dominant.

***Proof*** According to Equation (14), when *N*≥2, multiply *h*_1_ by (*N* − 1) to get: ${h_{1}}^{*}=\left(f_{1}-g_{1}\right)\cdot \left(N-1\right)=\eta D_{f}+\left(-qC+F+\eta D_{e}\right)\cdot \left(N-1\right)>0$. The sign of *h*_1_is the same as the sign of ${h_{1}}^{\prime }$, so *h*_1_ > 0. According to Equation (15), multiply *h*_*N* − 1_ by (*N* − 1) to get: ${h_{N-1}}^{*}=\left(f_{N-1}-g_{N-1}\right)\cdot \left(N-1\right)=\eta D_{e}+\left(-qC+F+\eta D_{f}\right)\cdot \left(N-1\right)>0$, so *h*_*N* − 1_ > 0. The drug quality improvement strategy gradually becomes a stable evolution solution.

Proposition 3 shows that when the penalty for choosing the drug cost reduction strategy is higher than the drug quality improvement cost, the pharmaceutical enterprises tend to choose the drug quality improvement strategy. When the loss caused by the drug cost reduction strategy is relatively high, pharmaceutical enterprises take the initiative to avoid risks and choose to produce high quality drug to obtain stable returns.

**Proposition 4** When *F* + *ηD*_*e*_ − *qC* < 0 and *F* + *ηD*_*f*_ − *qC* < 0, there is a threshold $N_{b}=\min\left(\frac{\eta D_{f}+qC-F-\eta D_{e}}{qC-F-\eta D_{e}},\frac{\eta D_{e}+qC-F-\eta D_{f}}{qC-F-\eta D_{f}}\right)$. When *N* ≤ *N*_*b*_, *h*_1_ > 0 and *h*_*N* − 1_ > 0are obtained, and the drug quality improvement strategy gradually becomes a stable evolution solution. There is another threshold $N_{c}=\max\left(\frac{\eta D_{f}+qC-F-\eta D_{e}}{qC-F-\eta D_{e}},\frac{\eta D_{e}+qC-F-\eta D_{f}}{qC-F-\eta D_{f}}\right)$, When *N*≥*N*_*c*_, *h*_1_ < 0 and *h*_*N* − 1_ < 0 are obtained, and the drug cost reduction strategy gradually becomes a stable evolution solution.

***Proof*** According to Equation (14), when *F* + *ηD*_*e*_ − *qC* < 0, multiply *h*_1_ by (*N* − 1) to get ${h_{1}}^{*}=\left(f_{1}-g_{1}\right)\left(N-1\right)=\eta D_{f}+\left(-qC+F+\eta D_{e}\right)\left(N-1\right)$. We take the first derivative of ${h_{1}}^{*}$ with respect to *N*, we can get ${\left({h_{1}}^{*}\right)}^{\prime }=-qC+F+\eta D_{e}<0$. The function *h*_1_ is monotonically decreasing. By the same token, the function *h*_*N* − 1_ is monotonically decreasing. Let *h*_1_ = 0 and *h*_*N* − 1_ = 0, we can get $N=\frac{\eta D_{f}+qC-F-\eta D_{e}}{qC-F-\eta D_{e}}$ and $N=\frac{\eta D_{e}+qC-F-\eta D_{f}}{qC-F-\eta D_{f}}$. When *N*≥*N*_*b*_, both *h*_1_ > 0 and *h*_*N* − 1_ > 0 are true; when *N* ≤ *N*_*c*_, both *h*_1_ < 0 and *h*_*N* − 1_ < 0 are true.

Proposition 4 shows that, When the number of pharmaceutical enterprises is lower than the threshold *N*_*b*_, pharmaceutical enterprises choose drug quality improvement strategy to improve market competitiveness. When the number of pharmaceutical enterprises is higher than the threshold *N*_*c*_, the pharmaceutical enterprises choose the drug cost reduction strategy.

## 6. Simulation analysis

In order to show how pharmaceutical enterprises choose the drug quality improvement strategy and drug cost reduction strategy under different conditions, *MATLAB 2021b* is used to simulate the strategic choice process.

In the sampling inspection of pharmaceutical preparations in Shandong Province, China, the “visible foreign body” item of an injection produced and sold by S Company did not meet the national drug standards, and this batch of injection was judged to be an inferior drug. S Company immediately started the product recall procedure and recalled a total of 17,000 boxes of injections. The total price of the recalled drug was 52,500 CNY and the sales income was 106,600 CNY. Taking 17,000 boxes of injection as an example, combined with the above case analysis, the minimum input cost *C* for production is 52,500 CNY, and the income *R* from drug sales is 106,600 CNY. In the first quarter of 2021, the incident was exposed on the Internet, which caused a sensation of public opinion. According to the annual report of S Company, the enterprise's operating income in the second quarter of 2021 decreased by 16.4% compared with the first quarter of 2021. Assuming that the sales return of the drug also decreased by 16.4% due to new media reports, it can be concluded that the negative effect *D*_*f*_ due to quality problems is 17,500 CNY. If S Company chooses to improve the injection quality, the positive effect *D*_*e*_ on the enterprises after the new media reports is 30,000 CNY. Government departments review and disclose the drug quality, and impose penalties on pharmaceutical enterprises that violate supervisions. Assume that the penalty fluctuates between 10–100% of income.

### 6.1. Drug quality strategy simulation under the dominance of external factors

**Figure 1:**
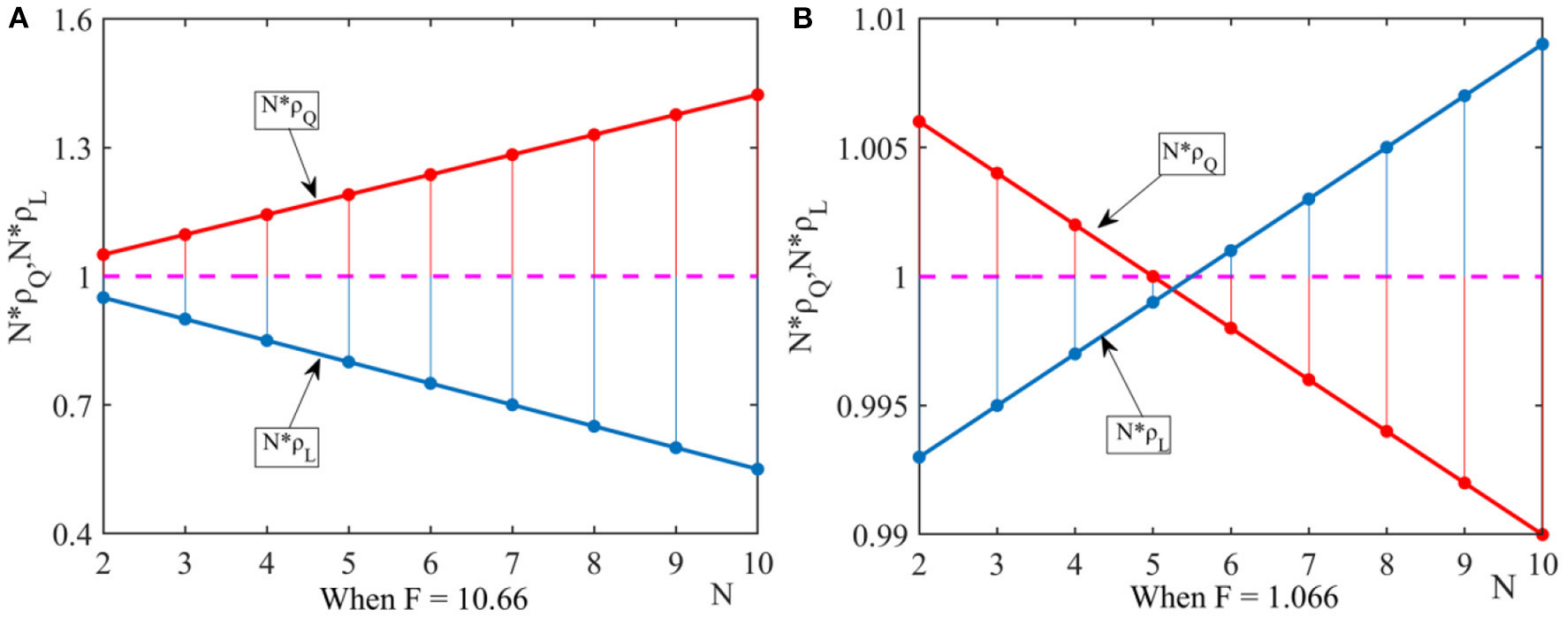
Effect of the number of pharmaceutical enterprises on fixed point probability. This figure shows that under the dominance of external factors, the effect of the number of pharmaceutical enterprises on fixed point probability when considering different government penalty. **(A)** When *F* = 10.66, **(B)** When *F* = 1.066.

(1) Under the weak selection condition, we let ξ = 0.01, *q* = 0.6, η = 0.9. In order to satisfy the conditions of Proposition 1 and Proposition 2, we let F = 10.66 and F, and obtain the fixed-point probabilities *N*×ρ_*Q*_ and *N*×ρ_*L*_ as shown in Figure 1.

It can be seen from Figure 1A that when government departments' penalties are high, the drug quality improvement strategy dominates in the group. It can be seen from Figure 1B that when government departments' penalties decreases, the number of pharmaceutical enterprises in the group has a threshold. When *N*∈(2, 5), pharmaceutical enterprises tend to choose the drug quality improvement strategy. When *N* > 5, pharmaceutical enterprises tend to choose the drug cost reduction strategy.

**Figure 2:**
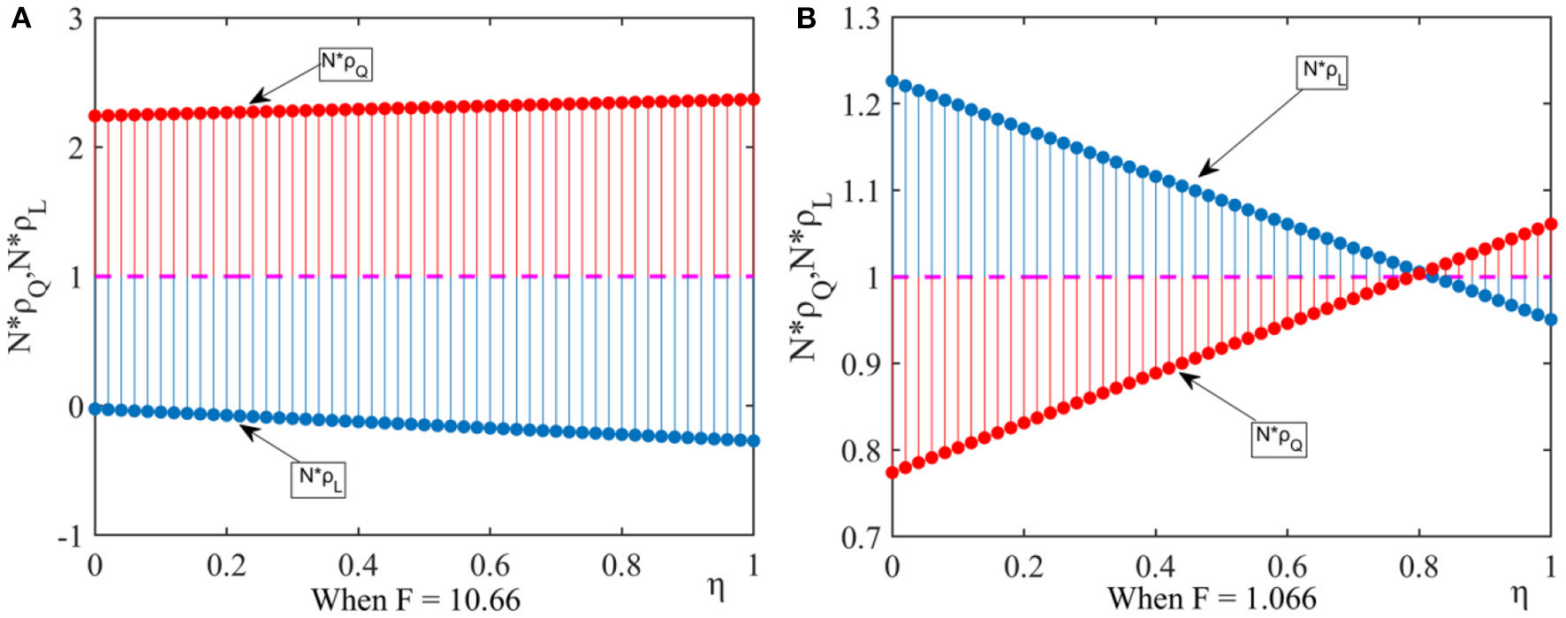
Effect of new media influence on fixed point probability. This figure shows that under the influence of external factors, the effect of new media influence on fixed point probability when considering different government penalty. **(A)** When *F* = 10.66, **(B)** When *F* = 1.066.

(2) We let ξ = 0.01, *q* = 0.04, *N* = 30. By comparing *F* = 10.66 with *F* = 1.066, we analyze the impact of the new media influence on the pharmaceutical enterprises' strategic choice. According to Figure 2, we can get the following results.

When the punishment of the government department is high, there are *N*×ρ_*Q*_ > 1 and *N*×ρ_*L*_ < 1, and the drug quality improvement strategy is dominant. When the punishment of the government department is low, if the new media influence is high, there are *N*×ρ_*Q*_ > 1 and *N*×ρ_*L*_ < 1, the drug quality improvement strategy is dominant. If the new media influence is low, there are *N*×ρ_*Q*_ < 1 and *N*×ρ_*L*_ > 1, the new media reports have little impact on pharmaceutical enterprises and the drug cost reduction strategy is dominant.

(3) Let ξ = 0.01, *q* = 0.04, and *N* = 30. We analyze the impact of government penalties*F*and new media influenceη on the strategic choice.

**Figure 3:**
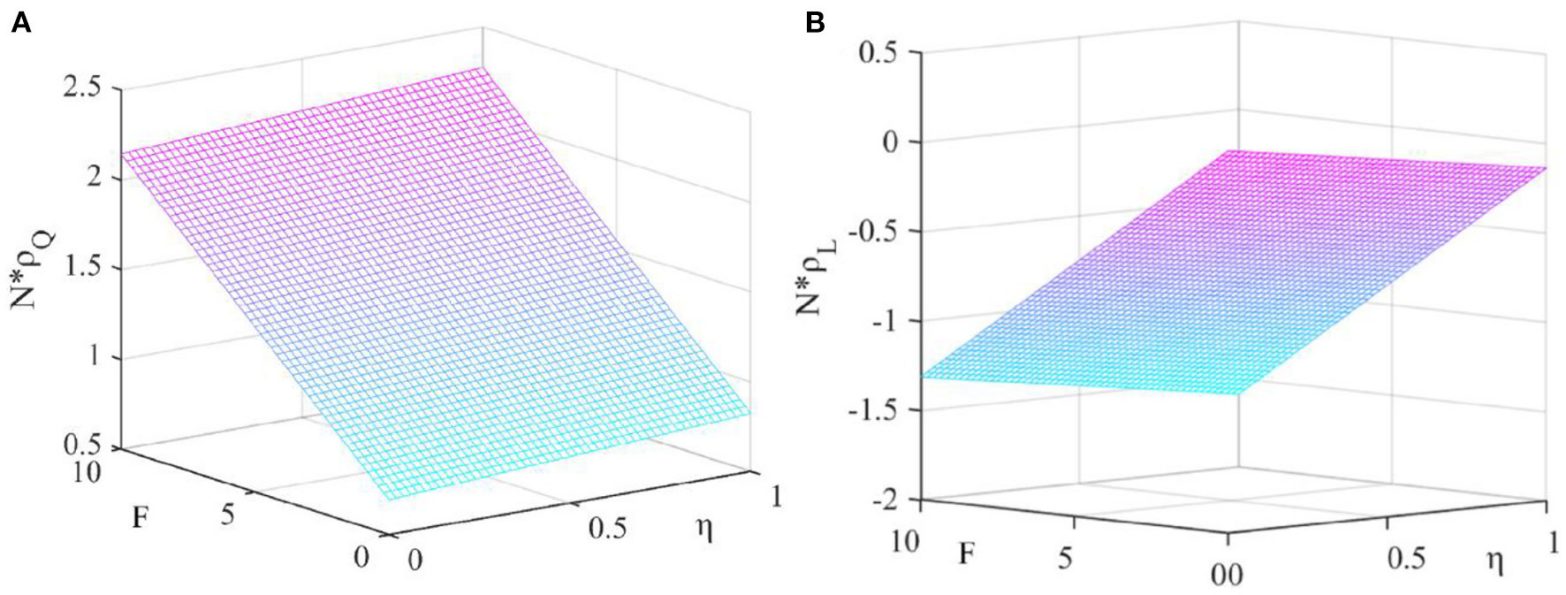
Effect of government penalty and new media influence. This figure shows the effect of the government penalty and the new media influence on the fixed-point probability under the dominance of external factors. **(A)** Fixed point probability *N*×ρ_*Q*_, **(B)** Fixed point probability *N*×ρ_*L*_.

It can be seen from Figure 3 that *N*×ρ_*Q*_ increases with the increase of *F* and η, *N*×ρ_*L*_ decreases with the increase of *F* and η. This shows that both the amount of government penalties and the level of new media influence are conducive to promoting pharmaceutical enterprises to produce high-quality drug.

### 6.2. Drug quality strategy simulation under the dominance of expected returns

**Figure 4:**
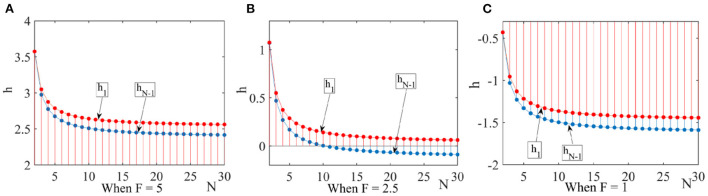
Effect of the number of pharmaceutical enterprises on strategic choice. This figure shows that under the dominance of expected returns the effect of the number of pharmaceutical enterprises on the strategic choice when considering different government penalty. **(A)** When *F* = 5, **(B)** When *F* = 2.5, **(C)** When *F* = 1.

(1) Low expected revenue is one of the important reasons for pharmaceutical companies to produce low-quality drugs. In order to satisfy the conditions of Proposition 3 and Proposition 4, we let η = 0.6, *q* = 0.7, and *F* = { 5, 2.5, 1}. We can obtain the images of *h*_1_ and *h*_*N* − 1_ as shown in Figure 4.

When the penalties for producing drug are higher than the quality improvement cost, *h*_1_ > 0 and *h*_*N* − 1_ > 0, the group is favorable for the drug quality improvement strategy to invade the drug cost reduction strategy. When the sum of the penalties for producing drug plus the negative effects is still less than the drug quality improvement cost, pharmaceutical enterprises are more inclined to choose drug cost reduction strategy. It can be seen that pharmaceutical enterprises make strategic choice according to expected returns.

**Figure 5:**
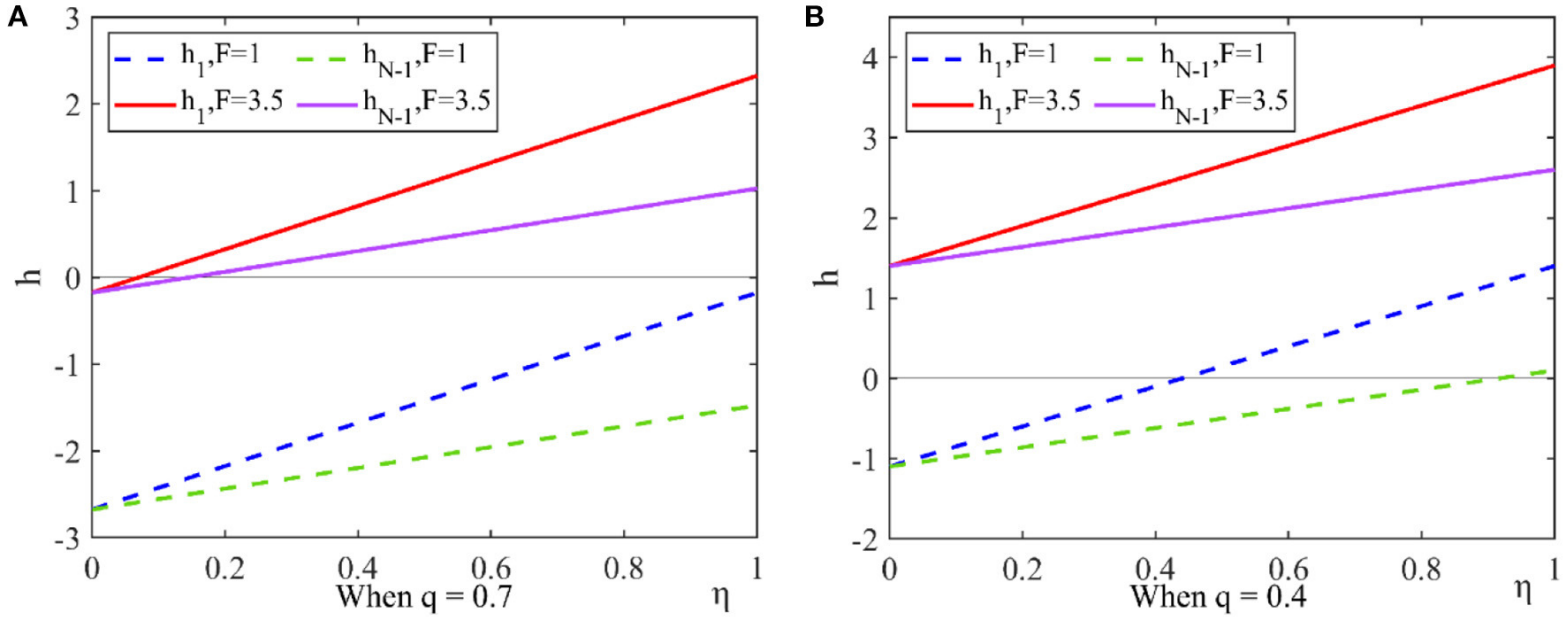
Effect of government penalty and new media influence on strategic choice. This figure shows the effect of government penalty and the new media influence on the choice of drug quality improvement strategy under the dominance of expected returns. **(A)** When *q* = 0.7, **(B)** When *q* = 0.4.

(2) Under the condition of strong selection, the drug quality improvement cost, the new media influence and the government penalty all have an impact on the strategic choice. We let *N* = 30, *q* = {0.7, 0.4}, *F* = {1, 3.5}, and analyze the strategic choice in different conditions. According to Figure 5, we can get the following results.

When the drug quality improvement cost and the government penalty are high, some pharmaceutical enterprises choose the drug quality improvement strategy in order to avoid risks. When the drug quality improvement cost and the government penalty are low, the new media influence has an impact on the pharmaceutical enterprises' strategic choice. When the new media influence is higher than a threshold, pharmaceutical enterprises choose drug quality improvement strategy.

## 7. Discussions

This paper studies the evolution process of the pharmaceutical enterprises' drug quality strategic choice. Each pharmaceutical enterprise can choose two strategies: drug quality improvement strategy and drug cost reduction strategy. Based on the *Moran process*, a fitness linear function expression is constructed to describe the expected returns of pharmaceutical enterprises. We analyze how pharmaceutical enterprises make strategic choice when external factors dominate and expected returns dominate. Finally, *MATLAB 2021b* is used to simulate the strategic choice process under different conditions. This paper provides suggestions for the quality strategic choice of pharmaceutical enterprises with the participation of new media under the government supervision. The main suggestions are as follows.

Firstly, pharmaceutical enterprises should increase investment in research and development, comprehensively optimize production activities, and improve the competitiveness of enterprises. By improving the technical level, pharmaceutical enterprises can promote the drug quality.

Secondly, government departments should strengthen the awareness of quality responsibility, conduct random sampling inspections of drug, and disclose sampling inspection information in a timely manner. By setting up a reasonable reward and punishment system, government departments punish enterprises that violate supervisions.

Thirdly, the new media should strictly verify the authenticity of the information before reporting, in order to prevent public opinion from publishing false information. The new media timely report the illegal production of enterprises, refuse to collude, and increase their own influence.

Finally, government departments should improve the social responsibility awareness of new media and strengthen the supervision of new media. By penalizing new media that publish false information, government departments promote truthful reporting by new media.

## 8. Conclusions

Drug quality is directly related to the people health, so it is important to improve the drug quality and strengthen the management of drug quality. This paper explores the Moran process of drug quality strategic choice of pharmaceutical enterprises considering the participation of new media and government department. By studying the evolution trajectory of drug quality decisions of a limited number of pharmaceutical enterprises, the conditions for pharmaceutical enterprises to choose different strategies are obtained.

The research found that: first of all, when the government penalty is high, pharmaceutical enterprises choose drug quality improvement strategy to avoid risks. Secondly, when new media participates in supervision, the higher the influence of new media, the more pharmaceutical enterprises choose the drug quality improvement strategy. Thirdly, when the quality cost factor is low and the government penalty is high, the drug quality improvement strategy is dominant. Finally, when the nature of the industry determines that there are a large number of pharmaceutical enterprises, pharmaceutical enterprises that choose the two strategies coexist in the industry.

In future research, we can further expand that the strategic choice is divided into multiple, and the external factors that affect the strategic choice can be subdivided by introducing random processes.

**Proof of Equation 11 and Equation 12**

Let *a* = *R* − (1 + *q*)*C*, *b* = *R* − (1 − *q*)*C* + *ηD*_*e*_, *c* = *R* − *C* − *F* − *ηD*_*f*_, *d* = *R* − *C* − *F*.


$$\begin{array}{c} \frac{g_{i}}{f_{i}}=\frac{1-\xi +\xi \pi_{Q}^{i}}{1-\xi +\xi \pi_{L}^{i}}=1+\xi \left(\pi_{Q}^{i}-\pi_{L}^{i}\right)+o\left(\xi \right)\\ \;=1+\frac{\xi }{N-1}\left[i\left(b+c-a-d\right)+\left(a+Nd-d-Nb\right)\right]+o\left(\xi \right) \end{array}$$

According to Equation 9, we can get this.


$$\begin{array}{l} \frac{1}{\rho_{Q}}=1+\left(1+\frac{\xi }{N-1}[(b+c-a-d)+(a+Nd-d-Nb)]\right)\;\\ \;+\left(1+\frac{\xi }{N-1}[3(b+c-a-d)+2(a+Nd-d-Nb)]\right)\;\\ \;+\cdots \cdots \\ \;+(1+\frac{\xi }{N-1}\\ \left[\frac{N(N-1)}{2}(b+c-a-d)+(N-1)(a+Nd-d-Nb)\right])+o(\xi )\\ =N\left(1+\frac{\xi }{6}[N(a+2b-c-2d)-(2a+b+c-4d)]\right)+o(\xi ) \end{array}$$

$$\rho_{Q}\approx \frac{1}{N}\left(1+\frac{\xi }{6}[(a+2b-c-2d)N-(2a+b+c-4d)]\right)$$

$$\rho_{Q}\approx \frac{1}{N}+\frac{\xi }{6N}[(a+2b-c-2d)N+(-2a-b-c+4d)]$$

$$\rho_{L}\approx \frac{1}{N}+\frac{\xi }{6N}[(-2a-b+2c+d)N+(4a-b-c-2d)]$$

Let α = *a* + 2*b* − *c* − 2*d*, β = − 2*a* − *b* − *c* + 4*d*, γ = − 2*a* − *b* + 2*c* + *d*, δ = 4*a* − *b* − *c* − 2*d*.


$$\begin{array}{l} \;\rho_{Q}=\frac{1}{1+\sum_{k=1}^{N-1}\prod_{n=1}^{k}\frac{g_{i}}{f_{i}}}\approx \frac{1}{N}\text{+}\frac{\xi }{\text{6N}}\left(\alpha \text{+}N\beta \right),\;\rho_{L}=\\ \frac{1}{1+\sum_{k=1}^{N-1}\prod_{i=k}^{N-1}\frac{f_{i}}{g_{i}}}\approx \frac{1}{N}\text{+}\frac{\xi }{\text{6N}}\left(\gamma +N\delta \right). \end{array}$$

## Data availability statement

The original contributions presented in the study are included in the article/supplementary material, further inquiries can be directed to the corresponding author.

## Author contributions

YX wrote the manuscript, solved the models, and made data analysis. LZ designed the research question, constructed the models, revised, and edited the manuscript. All authors read and approved the manuscript.
